# Author Index for Volume 103

**DOI:** 10.1038/sj.bjc.6606038

**Published:** 2010-12-07

Aaltonen, LA 1875

Aarntzen, EA 1415

Aarts, MJ 1742

Abad, A 581

Abaid, LN 1657

Abajo, A 1529

Abdah-Bortnyak, R 542

Abecassis, J 715

Absolom, K 1485

Abulrob, A 1606

Abuzeid, WM 1822

Acquavella, J 947

Adair, JE 1182

Adamson, DJA 475

Adema, GJ 1415

Adenis, A 1349

Adonis, M 1277

Aerts, JGJV 629

A’Hern, R 291

Ahlbom, A 1128

Ahn, JS 845

Ahn, YC 845

Aitken, JF 1663

Al-Batran, S-E 1518

Albaghdadi, H 1606

Albanes, D 132, 1089

Albert, PS 1453

Alberti, S 1835

Alcasabas, AP 149

Algazi, A P 1548

Ali, M 201

Ali, R 143

Allal, BC 61

Allen, DS 90

Allen, M 607

Allen, NE 1755

Allgayer, H 802

Allmaier, G 1201

Almstrup, K 1269

Alonso, MM 1292

Alos, L 510

Alvarez-Suarez, S 1524

Amarasinghe, HK 303

Amatori, S 239

Ambrogi, F 1835

Ameziane, N 29

Amiot, M 1808

An, Y 567

Ananda, S 1885

Andaya, A 1097

Anderson, JH 1356

Anderson, M 676

Anderson, WF 1081

Andersson, E 1255

Andersson, M 575

Ando, M 297

Ando, T 552

Andreopoulos, D 925

Andreotti, G 115

Andrews, S 1548

Andrulis, IL 1103

Angelelli, B 324

Angenendt, M 649

Ansell, SM 1783

Anton, J 209, 928

Aparicio, S 668, 1137

Appleyard, V 232

Araki, K 1822

Aranda, E 581

Arbabi, M 1606

Ardern-Jones, AT 918

Arenas, RB 486

Arizumi, T 1644

Arkenau, H-T 332

Armand, JP 993

Armstrong, BK 1510

Arnould, L 1335

Arra, C 1680

Arya, AK 186

Ash, S 1597

Ashfield, A 475

Ashley, AM 910

Ashworth, A 1192, 1760

Askenasy, N 1597

Aslam, MA 186

Asomaning, K 423

Assayag, F 1192

Atwood, WJ 885

Aubele, M 663

Auer, G 663, 1706

Augustin, HG 1407

Avallone, A 1680

Avenin, D 560

Ayoub, C 715

Azodi, M 812

Baade, PD 1663

Baay, M 1627

Baba, Y 1025

Babur, M 201

Bacchetti, S 324

Bachleitner-Hofmann, T 1201

Baens, M 265

Bagaloni, I 239

Baglietto, L 411, 668, 1137

Bago-Horvath, Z 622

Bai, M 265

Bailey, A 209, 928

Baker, JHE 52

Baker, L 232, 475

Baker, R 1034

Bakken, K 1755

Baldini, C 772

Balmar, SM 870

Bandrés, E 1292, 1529

Banerji, U 1149

Banks, RE 101, 1649

Barbachano, Y 918

Bargou, R 196

Barnes, I 143

Barnetson, R 1875

Baron, JA 947

Barrett, J 454

Barrett, JH 1229

Barrett, M 649

Barricarte, A 1755

Bartlett, JMS 663

Bartsch, R 1201

Basu, S 1221

Bataille, R 1808

Batt, L 1801

Baumann, S 1201

Baumann, W 1163

Baxter, RC 391

Beane Freeman, LE 1081

Bedard, PL 1788

Beddows, S 209, 928

Beerblock, K 560

Beiss, B 1518

Belaud-Rotureau, M-A 1698

Bello, L 827

Bellone, M 812

Bellone, S 812

Benavides, M 581

Benchimol, S 1432

Bengala, C 1019

Benítez, H 1277

Beral, V 143

Beretov, J 1008

Berg, P 1489

Bergen, AW 1870

Berger, W 622

Bergmann, M 1201, 1755

Berkhof, J 939

Bernaudin, J-F 560

Bernhard, J 1318

Bernier, J 1173

Berns, B 1149, 1154

Berns, EM 765

Berrino, F 1755

Bertani, G 827

Bertazzi, PA 1870

Bertolini, F 1019

Berton, A 930

Bertucci, F 482

Betka, J 1173

Bettelli, S 1019

Bézieau, S 1875

Bhardwaj, A 1671

Biganzoli, E 1835

Bijl, MJ 765

Billan, S 542

Bishop, DT 1229

Bishop, H 94

Bitarte, N 1529

Blair, A 727

Blanco, I 1875

Blay, J-Y 165, 485

Blettner, M 931

Bloomer, WD 201

Blows, FM 668, 1137

Bobe, G 1453

Boddy, AV 1588

Bodet, L 1808

Bodmer, WF 340

Bodoky, G 1318

Boeckx, C 1627

Boeing, H 1755

Boezen, HM 685

Bohndiek, SE 1400

Boissin, J-L 1115

Bomken, S 439

Bonassi, S 885

Bone, EA 1362

Boni, V 1529

Bonnetain, F 1335

Bonneterre, J 772

Bonomi, P 1221

Book, K 1489

Boracchi, P 1835

Bordignon, C 837

Bordoni, A 416

Borgia, JA 1221

Borgquist, S 1755

Born, O 1548

Bost-Bezeaud, F 1115

Botsivali, M 1749

Bottomley, A 1173

Bouchardy, C 416

Bourantas, K 265

Bourhis, J 347

Boutron-Ruault, M-C 1755

Bouville, A 1115

Boven, E 757, 993

Bower, M 265

Boyd, MT 186

Bozzarelli, S 837

Bracht, K 340

Bradley, G 1432

Bragard, I 171

Branston, L 1801

Braselmann, H 663

Brassart Pasco, S 1562

Brasso, K 1496

Breaker, K 796

Brega, NM 993

Brenner, H 149

Brenton, JD 1710

Briassoulis, E 1048

Briggs, AH 776

Brindel, P 1115

Brindle, KM 1400

Bringuier, P-P 165, 482

Brinton, LA 1097

Broderick, P 1875

Brodie, AMH 1001

Brough, R 1192

Brown III, JV 1657

Broza, YY 542

Brunetto, AT 607

Brunner, EJ 747

Brunton, VG 899, 1831

Bruzzese, F 1680

Büchler, P 1571

Buczkowski, AK 52

Budillon, A 1680

Buecher, B 1875

Bueno-de-Mesquita, HB 1755

Buffa, FM 929, 1136

Buhard, O 1840

Buhl-Jensen, P 12

Bui, B 482

Bullock, S 918

Burchill, SA 1380

Burdette, L 1870

Burge, M 597

Burger, DM 1637

Burger, H 987

Burghuber, C 1201

Burley, VJ 747, 1749

Burris, HA 1783

Burwinkel, B 1237

Butow, PN 1502

Butturini, G 391

Buys, TPH 1277

Buza, N 812

Byloos, E 1815

Byrnes, G 1755

Caballero, M 510

Cade, JE 747, 1749

Cagol, PP 324

Cai, H 567, 1085

Cai, W 1369

Cairns, BJ 747

Cairns, DA 101

Calasanz, MJ 1292

Caldas, C 668, 1137

Caldwell, GM 910

Caligaris-Cappio, F 837

Callagy, G 668, 1137

Calmy, A 416

Campagnolo, P 1422

Campanella, C 1542

Campbell, H 575, 1875

Campbell, HE 776

Campieri, M 975

Canal, P 61

Candamio, S 1536

Canon, J-L 1815

Cantarelli, B 1562

Cantley, LC 1025

Capellà, G 1875

Caporali, A 1422

Caporaso, NE 727, 1870

Caradec, J 591, 1475

Carafa, V 382

Cardesa, A 510

Cardis, E 1115

Cardoso, F 1788

Carozza, SE 136

Carpanese, L 324

Carrato, A 581

Carrera, M 510

Cartledge, J 1649

Casagrande, F 812

Caselles, O 61

Cassidy, A 423

Cassier, PA 165

Castelbuono, DJ 1588

Cavassini, M 416

Cavenagh, J 156

Cawthorne, C 201

Cejka, D 622

Cellier, D 165

Cetin, K 947

Chajes, V 1755

Cham, KKY 52

Chan, AT 1025

Chan, DA 1

Chan, M 1229

Chan, S 1794

Chandran, PA 693

Chang, C-WA 52

Chang, P-L 852

Chang-Claude, J 1755

Chao, Y 954, 1343

Chapman, RS 727

Charbonneau, C 1307

Charles, P 291

Chatterjee, S 475

Chazono, H 877

Chen, BE 727

Chen, C-A 1773

Chen, C-J 1773

Chen, C-S 852

Chen, H 1008

Chen, J-S 1343

Chen, K 1085

Chen, L-T 954, 1343

Chen, P-J 954

Chen, R 1518

Chen, S-C 954

Chen, Y 256

Cheney, R 1066

Cheng, YC 1331

Chevreau, C 482

Chia, YH 759

Chianese, MI 1680

Chilosi, M 1422

Chirlaque, M-D 1755

Cho, EY 845

Chong, H 1229

Chopitea, A 1529

Chosa, M 1580

Choudhary, AK 1391

Chow, EJ 136

Chow, I H-I 1773

Christensen, BC 885

Christensen, J 730

Christiani, D 423

Chu, EM 52

Chu, T 446

Chu, T-Y 1773

Chuang, C-K 852

Chuang, CP 362

Chung, L 391

Chung, SW 52

Citterio, G 837

Clark, J 1858

Clark, JR 1510

Clavel-Chapelon, F 1755

Cleary, SP 1875

Cleator, S 1858

Clifford, GM 416

Clifford, SC 1588

Clive, S 617

Closset, J 930

Cocco, E 812

Coebergh, JWW 1462, 1742

Coens, C 1173

Cognetti, F 1542

Cohen, RJ 411

Colas, C 1840

Colburn, NH 1453

Coleman, MP 446

Collura, A 1840

Colombi, S 837

Conchillo, A 1292

Conroy, T 1349

Conte, PF 1019

Conway, C 1229

Cook, M 1229

Cook, MB 1467

Cooke, R 1729

Coon, JS 1221

Coradini, D 1835

Corcoran, NM 462

Cosimelli, M 324

Costa, LJ 796

Costello, AJ 462

Costelloe, CM 927

Cottu, P 1192

Couderc, B 61

Coudert, B 1335

Coulthard, A 597

Countouriotis, AM 993

Coupe, VMH 939

Coupland, VH 1076

Courbon, F 61

Coutelle, O 1407

Cowell, JK 1066

Cozzi, PJ 1008

Cree, IA 656

Cremer, B 1407

Crespi, CM 1128

Crighton, F 796

Cristobal, I 1292

Croitoru, EM 1875

Cronin-Fenton, DP 947

Crook, T 265

Cross, AJ 1453

Crowley, E 12

Cubitt, A 597

Cui, W 1369

Cumming, A 73

Cummings, J 1313

Cummings, NM 1710

Cupissol, D 482

Curtin, NJ 1588

Curtis, RE 1081

Cuzick, J 94

Czuczman, MS 1783

Daemen, T 685

Dahiya, R 256

Dahm, CC 747

Dai, C 567

Daigo, Y 217

Dal Maso, L 416

Dalén, J 575

Damato, B 285

D’Amelio Jr, AM 423

Daniel, RA 1588

Darnton, AJ 430

Daud, AI 1548

Davies, D 1692

Davies, EA 1076

Davies, SM 1724

Davis, H 1154

Davis, ML 1331

Dawson, S-J 668, 1137

de Boer, A 1415

de Boer, C 1149

de Bono, JS 12, 332, 607

De Braud, FG 837

de Cremoux, P 1192

de Graan, A-JM 765

de Jong, S 685

de Jonge, M 1362

de Jonge, MJA 987

de Kok, TM 1749

de la Cámara, J 1536

De la motte Rouge, T 347

de Plater, L 1192

de Reyniès, A 715

de Ruggiero, I 1680

de Silva, N 209, 928

de Vathaire, F 1115

de Vries, IJM 1415

de Witte, PAM 178

Dearnaley, DP 918

Debelle, L 1562

Decaestecker, C 930

Decaudin, D 1192

DeConti, RC 1548

Decouvelaere, A-V 165

Degardin, M 1173

Degerman, S 1255

Dehler, S 416

Del Giovane, C 1019

Delord, J-P 61

Delprado, WJ 1008

Delvaux, N 171

Demers, L 332

Demetter, P 930

Demeyere, TBJ 1462

Deng, G 256

Depenni, R 1019

Deray, G 1815

Desar, I 1362

Desar, IME 1637

Descamps, G 1808

Deschoolmeester, V 1627

Desvaux, C 1606

Deutsch, E 347

Devière, J 930

Devling, T 186

Devy, J 1562

Dewar, JA 475

Di Cintio, A 1680

Di Fiore, F 1765

Di Gennaro, E 1680

Di Molfetta, S 975

Diaz, A 510

Dickinson, I 73

Diebold, I 1571

Dietl, J 693

Dietrich, D 1318

Dimitrakopoulou-Strauss, A 196

Dinjens, WNM 1840

Dinkel, A 1489

Diodoro, MG 324

Dirix, LY 532

Dite, GS 1103

Dive, C 1313

Dobbins, TA 1510

Doki, Y 1617

Dolley-Hitze, T 1698

Dominek, P 1422

Donadoni, G 837

Donehower, R 649

Donze, J 498

Dores, GM 1081

Dörken, B 505

Dorronsoro, M 1755

Dorssers, LCJ 1284

Dorvault, N 347

Doyon, F 1115

Drabkin, H 796

Draper, GJ 1122, 1128

Drew, Y 1588

Driouch, K 524

Driver, KE 668, 1137

Drozdovitch, V 1115

Dubois-Marshall, S 401

Duca, L 1562

Ducimetière, F 165

Duck, L 1815

Dudderidge, TJ 701

Duffy, SW 423

Dufresne, A 482

Duggan, C 94

Dummer, R 820

Duniho, S 676

Dunlop, MG 575, 1875

Duval, A 1840

Dzieȩgiel, P 524

Easton, DF 918

Eccles, RM 186

Edeline, J 1698

Eder, IE 1479

Edler, D 575

Edler, L 820

Edmonds, K 1149

Edward, M 401

Edwards, J 899, 1831

Edwards, SM 918

Eechoute, K 757

Eeles, RA 918

Egeberg, R 730

Eguchi, H 1617

Eichenmüller, M 43

Ekelund, S 575

El-Fert, A 186

Eleftheraki, AG 1048

Eling, TE 1182

Elliott, F 1229

Elliott, RA 315, 1887

Ellis, I 668, 1137, 1794

Ellis, IO 94

Ellis, L 446

Ellis, MJ 759

Ellis, NA 1875

Elsberger, B 899, 1831

Elzi, L 416

Emanueli, C 1422

Emrich, M 1489

Endo-Munoz, L 73

Engel, JB 693

Engelholm, S-A 12

Engelman, JA 1025

Engels, EA 112

Engi, H 178

English, DR 411

Enholm, S 1875

Escudier, B 1309

Eskens, FALM 987, 1362

Ess, S 416

Etienne, A-M 171

Etienne, PL 1349

Ettore, F 1335

Ettorre, GM 1542

Etzel, CJ 423

Evans, DGR 918

Evans, EE 401

Exon, D 232

Eyholzer, M 275

Faber, LP 1221

Fajac, A 560

Falconer, A 918

Falk, RT 1097

Fallowfield, L 454, 1801

Fan, J-W 961

Fanelli, M 239

Faratian, D 401

Farewell, D 1801

Farewell, V 454, 1801

Farlow, EC 1221

Farrington, SM 575, 1875

Faupel-Badger, JM 1097

Favier, L 1335

Fehrmann, R 607

Fehrmann, RSN 685

Feltbower, RG 1448

Fentiman, IS 90, 94, 747

Fenwick, J 186

Ferguson, TR 1149

Ferretti, S 1835

Feuchtinger, A 663

Field, JK 423

Figdor, CG 1415

Figueroa, JD 1097

Filipits, M 622

Fiore, F 324

Fiore, N 1736

Fišer, K 439

Fisher, C 918

Flaig, TW 796

Fleissig, A 454

Flejou, J-F 1840

Fleming, IN 1391

Flexman, JA 52

Flockhart, DA 291

Floren, L 1548

Foekens, JA 1284

Fong, PC 332

Fontaine, J-J 1192

Fontana, A 1019

Forbes, JF 94

Forbes, NS 486

Forman, D 446

Formica, M 239

Forrest, MS 918

Foulis, AK 870

Fountzilas, G 1048

Fournier, A 1755

Fox, EE 136

Fox, NL 1783

Franceschi, S 157, 416

Francesconi, AB 597

Franco, R 1680

Fraser, LK 1749

Frasson, C 1835

Fratto, ME 1305

Fraumeni Jr, JF 115, 727, 1081

Frebourg, T 1765

Frederiksen, BL 1496

Friberg, E 127

Frick, H 416

Friess, H 1571

Fryzek, JP 947

Fu, S 1215

Fuchs, CS 1025

Fujii, H 552

Fujimura, L 877

Fujiwara, T 370

Fujiwara, Y 297

Fukao, A 1443

Fukuoka, M 36, 354

Fukushi, J-i 370

Fukutomi, A 469

Fullerton, R 899

Fülöp, F 178

Fumoleau, P 1335

Funai, J 469

Funakoshi, A 469

Furrer, H 416

Furuse, J 469

Fusi, V 239

Gabriel, FG 656

Gallagher, FA 1400

Gallegos, I 1277

Gallinger, S 1875

Galluzzo, S 1305

Gandy, R 391

Gao, L 1085

Gao, Y-T 115

García, MA 1292

García-Alfonso, P 1524

Garcia-Closas, M 1097

Garcia-Foncillas, J 1529

García-Ortí, L 1292

Garrido-Laguna, I 649

Garufi, C 1542

Gasperini, D 324

Gatter, KC 1209

Gaudet, MM 1097

Gauthier, M 1335

Geatti, O 324

Gee, GV 885

Gehr-Swain, BN 918

Geinitz, H 1489

Gelderblom, H 757

Gelmon, K 668, 1137

George, WD 94

Gerlinger, M 1139

Ghazi, S 575

Giaccia, AJ 1

Giampalma, E 324

Giatromanolaki, A 1209

Gibbs, P 1885

Gierach, GL 933

Giese, N 1571

Gil, L 1277

Giles, GG 411, 668, 1103, 1137

Ginés, A 581

Giorgi, L 239

Giovannucci, E 1025

Glaser, A 1448, 1485

Glaysher, S 656

Gligorov, J 560

Glimelius, B 1318

Gloesenkamp, C 18

Gnant, M 1201

Godinho, MFE 1284

Goede, V 1407

Goldenberg, DM 82

Goldin, R 1858

Goldstein, BH 1657

Golfieri, R 324

Goloubeva, O 1001

Gómez-Benito, M 1292

Gomez-Bougie, P 1808

Gomułkiewicz, A 524

Goncalves, A 482

González, CA 1755

Gonzalez-Angulo, AM 1331

Gooden, MJM 685

Gordon, KA 676

Gore, ME 1149

Görlach, A 1571

Graf, A 1201

Grande, C 1536

Gray, AM 776

Green, AC 1663

Green, AR 668, 1137

Green, MC 1331

Green, SR 1391

Greenwood, DC 747, 1749

Gregory, W 1649

Greil, R 1201

Griffin, B 265

Griffin, JL 1297

Griffin, M 597

Griffoni, C 975

Grimm, C 613

Gröne, J 505

Gualberto, A 332

Guchelaar, H-J 757

Guerrero, J 1040

Guiu, B 1335

Guiu, S 1335

Güntner, M 1518

Guo, C 1085

Guo, F 1085, 1554

Gupta, A 708

Gustafson, DL 796

Guy, M 918

Guyader, C 1192

Gyémánt, N 178

H-Zadeh, M 1422

Haanen, JB 757

Hacker, UT 1407

Hagihara, J 1128

Haick, H 542

Haiman, CA 120

Haitel, A 622

Hakim, M 542

Hakim, S 510

Hakulinen, T 1109

Hall, AL 918

Hall, G 1846

Hallek, M 1407

Hallmans, G 1755

Halpern, W 1783

Hamamoto, R 217

Hamaoka, T 927

Hamelin, R 1840

Han, T-Q 115

Hanazawa, T 877

Hao, J 1008

Harbeck, N 1518

Harnden, P 1649

Harnett, GB 1510

Harper, S 437

Harris, AL 776, 929, 1136, 1209

Harrison, DJ 401

Hartog, V 993

Harutyunyan, N 1369

Hashimoto, M 889

Hassall, T 1663

Hassel, JC 820

Hasskarl, J 1237

Hatashita, E 36

Hatzimichael, E 265

Haubein, NC 861

Haugstetter, AM 505

Häusler, SFM 693

Hawkins, R 1154

Hayden, H 622

Hayes, DF 291

He, J 120

He, X 727

He, Z 1085

Heerema, NA 1724

Hefler, L 613

Hegmans, JPJJ 629

Hei, TK 1263

Heideman, DAM 939

Heidenreich, O 439

Hejmadi, RK 910

Hekmatyar, SK 1297

Hellemans, P 987

Hemmerlein, B 43

Henderson, BE 120

Hendriks, RW 629

Henne-Bruns, D 1163

Hennebelle, I 61

Henrich, G 1489

Henry, NL 291

Henson, MA 486

Heong, V 1885

Hepworth, SJ 1749

Herberger, B 622

Herbert, B-S 1716

Herrmann, R 1318

Herschbach, P 1489

Hess, V 1318

Hesselink, AT 939

Heuer, S 693

Hidalgo, M 649

Hilden, JM 1724

Hill, C 1115

Hines, OJ 1571

Hirakawa, K 249, 1182

Hirano, K 1644

Hiraoka, N 1057

Hiro, J 787

Hjern, F 575

Ho, J 1432

Hoang, HN 411

Hocevar, BA 1716

Hodgson, JT 430

Hoffmann, B 18

Höfler, H 663

Hofstra, RMW 1840

Hohenberger, P 196

Hollema, H 685

Holm, J 575

Holten, I 1496

Honda, T 1325

Hong, AM 1510

Hong, M 1263

Hönig, A 693

Hooftman, L 1362

Hoogsteden, HC 629

Hope, Q 918

Höpfner, M 18

Hopper, JL 411, 1103

Horel, S 136

Horgan, PG 870, 1356

Horiguchi, S 877

Hortobagyi, GN 927, 1331

Horvat, R 613

Hosgood, HD 727

Hostettler, I 382

Hostomsky, Z 1588

Houlston, R 1875

Housden, C 1076

Houterman, S 1742

Hovens, CM 462

Howat, WJ 1710

Howell-Jones, R 209, 928

Hsi, LC 1182

Hsing, AW 115

Hu, D-e 1400

Hu, Z 812

Hugonin, S 560

Huntsman, D 668, 1137

Hwang, J 1548

Iacobelli, S 1835

Iacobuzio-Donahue, C 649

Iezzi, G 382

Ihnen, M 1048

Iida, K 370

Ijichi, H 1644

Iles, MM 1229

Iltis, J 1115

Im, Y-H 845

Ino, Y 1057

Inoue, M 889

Inoue, Y 787

Iqbal, U 1606

Isayama, H 1644

Ishida, T 1443

Ishihara, T 223

Ishikawa, T 249

Iwamoto, Y 370

Iwasa, T 36

Izzo, F 324

Jacobs, I 454

Jagdev, SPK 1649

Jäger, E 1518

James, MG 1149

James, ND 1154

Janes, SM 1692

Janicijevic, M 1706

Jänicke, F 1048

Jansen, MT 1269

Janson, M 575

Janssen-Heijnen, MLG 1742

Janus, N 1815

Jelic, S 1173

Jenkins, MA 1875

Jenkins, V 1801

Jerez-Gilarranz, Y 1524

Jerome, N 1297

Jesudason, EC 593

Jhavar, SG 918

Ji, L 1369

Jimenez, M 482

Jimeno, A 649

John, EM 1103

Johnsen, NF 730

Johnson, KJ 136

Johnson, NW 303

Johnson, P 656

Johnston, S 607

Jones, CE 910

Jones, D 1510

Jones, L 1313

Jones, ME 1760

Jones, TM 186

Jordan, F 899

Jordan, LB 475

Jorge, M 1536

Jørgensen, A 1269

Jørgensen, T 1496

Joseph-Pietras, D 1562

Jouan, F 1698

Julyan, PJ 201

Jundt, G 416

Kaaks, R 1755

Kabiru, EW 1736

Kakizaki, M 1443

Kakugawa, Y 1443

Kalogeras, KT 1048

Kameda, Y 517

Kamel-Reid, S 1432

Kamura, S 370

Kan, SW 143

Kanai, Y 1057

Kappler, R 43

Karavasilis, V 332

Kashiwagi, S 249

Kashkar, H 1407

Kasparian, NA 1502

Katakami, N 6

Kato, A 223

Katsumata, N 297

Katz, E 401

Kauppinen, RA 1297

Kavanagh, D 1858

Kawaguchi, Y 552

Kawai, M 1443

Kawajiri, H 249

Kawakubo, K 1644

Kaye, SB 607

Ke, Y 1085

Keiser, O 416

Keller, A 693

Kelly, JD 701

Kelly, P 232

Kelsey, KT 885

Keogh, RH 747

Kerger, J 1815

Keshelava, N 1369

Kesselring, R 1245

Kets, CM 1840

Kettunen, MI 1400

Keumeugni, C 591, 1475

Key, TJ 1755

Khamly, K 1554

Khan, K 1822

Khatri, G 256

Khaw, K-T 747, 1755

Kheifets, L 1128

Khosravi, P 1524

Kikkawa, N 877

Kilkerr, J 454

Kim, AW 1221

Kim, FJ 796

Kim, J 1510

Kim, K 845

Kim, KH 845

Kim, KM 676

Kimura, F 223

Kiniwa, M 354

Kinoshita, T 297

Kinouchi, H 552

Kinsey, SE 1448

Kissin, M 1229

Kita, A 36

Kitchener, H 209, 928

Kiyono, T 889

Klein, J 1783

Kleinjans, J 1749

Klocker, H 1479

Knauer, M 1788

Knight, JA 1103

Knight, LA 656

Knoblauch, P 12

Kobayashi, S 1617

Kocak, I 1154

Koessler, T 1875

Kogure, H 1644

Koh, W-P 1093

Koike, K 1644

Koinuma, J 217

Koldewijn, EL 1462

Kolonel, LN 120

Kondo, S 469

Kondo, T 1325

Konings, IRHM 987

Kono, K 552

Konzelmann, I 416

Koopman, M 159

Körner, A 802

Kornmann, M 1163

Koshiji, M 469

Koshiol, J 1870

Koslowsky, TC 1407

Kostner, H 676

Kosuge, T 1057

Kote-Jarai, Z 918

Koukourakis, MI 1209

Koumantaki, Y 1755

Kouno, T 297

Koutoku, H 36

Koyama, T 1057

Kozlowski, P 52

Kränkel, N 1422

Kreuser, E-D 1163

Krikun, G 812

Krockenberger, M 693

Kroll, ME 1122

Kron, M 1163

Kuh, D 747

Kumaraarachchi, M 303

Kumm, E 1783

Kunitoh, H 6

Kuriyama, S 1443

Küry, S 1875

Kurzen, H 820

Kusunoki, M 787

Kuten, A 542

Kuwata, K 36

Kyo, S 889

Kyrtopoulos, SA 1749

La Sorda, R 1835

Ladoire, S 1335

Ladurner-Rennau, M 1479

Lafleur, MVM 29

Lagergren, J 735

Lagergren, P 735

Laheru, D 649

Laing, S 1729

Lalloo, R 303

Lam, S 1277

Lam, WL 1277

Lambers, MEH 629

Lambiase, A 837

Lamezec, B 1349

Lammers, LA 765

Lamont, D 232

Lampejo, T 1858

Lan, Q 727

Landi, MT 1870

Langdon, SP 401

Langridge, C 1801

Lanoy, E 112

Lanza, E 1453

Lardon, F 1627

Larkin, JMG 1149

Lascols, O 1840

Lassen, U 12

Lastoria, S 324

Lattanzio, R 1835

Laudico, A 149

Laugé, A 1192

Launay-vacher, V 1815

Law, C-L 676

Lawson, MH 1710

Layer, G 1229

Layos, L 581

Lazzarini, G 975

Le Cesne, A 482

Le Gouill, S 1808

Le Marchand, L 120

Le Pogamp, P 1698

Le Prisé, E 1349

Le Quesne, J 668, 1137

Le, LW 1432

Ledergerber, B 416

Lee, CS 1510

Lee, H-P 1093

Lee, K-D 1343

Lee, K-M 727

Lee, S 265, 845

Lee, SY 362

Leenstra, S 29

Leffers, N 685

Leifland, K 1706

Lein, M 18

Leitch, EF 1356

Leitzel, K 332

Lenander, C 575

Lenze, D 505

Leon, E 82

Lerbs, W 1518

Lerro, CC 1467

Lesterhuis, WJ 1415

Lett, GS 861

Leung, HY 701

Leung, S 668, 1137

Levi, F 416

Lévy, E 560

Lévy, P 560

Lewis, IJ 1448

Li, A 1215, 1255

Li, C-P 1343

Li, D 1822

Li, D-W 961

Li, J 401

Li, L 291

Li, Q 115

Li, Y 715, 1008, 1085

Liao, C-H 1773

Liao, L-Y 954

Liao, S-K 852

Libert, Y 171

Lickliter, JD 597

Lidereau, R 524

Liénard, A 171

Ligtenberg, MJL 1840

Lih, FB 1182

Lilja, H 708

Liloglou, T 1846

Lim, MK 741

Limame, R 532

Lin, C-C 954

Lin, P-C 1343

Lin, P-Y 1144

Linabery, AM 1724

Lindblom, A 575, 1875

Lindforss, U 575

Lindner, K 663

Link, K-H 1163

Linklater, KM 1076

Linn, SC 1788

Linnoila, RI 1870

Liptay, MJ 1221

Lipton, A 332

Lise, M 416

Lissowska, J 1097

Liu, F 1085

Liu, J 256

Liu, L 498

Liu, P 927

Liu, XS 595

Liu, Y 340, 1085, 1215

Ljungberg, B 1255

Lloyd, BH 186

Lobo, S 1229

Lockwood, CJ 812

Loda, M 1025

Loddenkemper, C 505

Loebinger, MR 1692

Löfgren, L 1706

Loft, S 730

Lokiec, F 560

Look, MP 1284

Loos, M 1571

López, R 1536

Lord, CJ 1192

Loric, S 591, 1475

Loriot, Y 347

Louwman, WJ 1462, 1742

Lowe, D 1846

Lowenthal, RM 1128

Lu, C 1085

Lu, C-H 1343

Lu, S-N 954

Lu, Y 735

Lubbe, S 1875

Luben, RN 747

Lubin, JH 727

Luciano, A 1680

Ludyga, N 663

Lugli, A 382

Lujan, B 510

Luk, SK 362

Lunardi, M 1835

Lund, E 1755

Lundqvist, N 575

Lunt, SJ 201

Luo, Y 1606

Luppi, G 1019

Lurkin, A 165

Lybaert, W 1815

Lynge, E 310

Ma, CX 759

Ma, WW 649

Macedi, E 239

Machiels, J-P 1815

Madeddu, P 1422

Madewell, JE 927

Madigan, MC 1008

Maicas, M 1292

Maida, Y 889

Mailliez, A 772

Maini, CL 324

Majchrzak, M 524

Majid, S 256

Major, JM 132, 1089

Makretsov, N 668, 1137

Malavasi, N 1019

Maldonado, NV 1369

Mallet, Y 772

Mallon, EA 899, 1831

Mancini, R 324

Manegold, C 196, 802

Mangialardi, G 1422

Manjer, J 1755

Mann, GJ 1502

Mantzoros, CS 127

Manzano, JL 581

Marangoni, E 1192

Marchal, S 171

Marchetti-Deschmann, M 1201

Marcotegui, N 1292

Marcuello, E 581

Maréchal, R 930

Marincola, FM 1870

Maringe, C 446

Marisa, L 715

Mårlind, J 827

Marsit, CJ 885

Marten-Mittag, B 1489

Martikainen, P 1109

Martin, B 1698

Martin, JC 1716

Martin, M 1524

Martínez, J 1040

Martinez, VD 1277

Martinez-Balibrea, E 581

Martínez-Cardús, A 581

Martiny, L 1562

Marubashi, S 1617

Maruyama, T 552

Marx, A 196

Maspoli, M 416

Massard, C 993

Massoner, P 1479

Massutí, B 581

Mathijssen, RHJ 757, 765

Matsubara, S 1644

Matsumoto, Y 370

Matsuoka, T 1182

Matthews, CE 933

Matthews, GM 910

Mauch, C 820

Mauer, ME 1173

Maughan, T 1801

Mayer, F 196

McArdle, CS 970

McCarron, J 597

McCredie, MRE 1103

McEarchern, JA 676

McGlynn, KA 1467

McGraw, M 498

McKee, RF 1356

McKinney, PA 1448, 1749

McLaughlin, CC 136

McLean, CA 668, 1137

McLoone, JK 1502

McMillan, DC 870, 970, 1356

McShane, LM 1870

Meehan, RR 401

Méeus, P 165

Meijer, CJLM 939

Meijer, D 1284

Meiser, B 1502

Meitz, J 918

Mekenkamp, LJM 159

Mele, V 382

Méndez, JC 1536

Menon, U 454

Ménoret, E 1808

Merckaert, I 171

Mern, DS 1237

Mesia, R 1173

Metzen, E 1571

Meunier, J 171

Meyerhardt, JA 1025

Mezei, G 1128

Miao, Y 567

Miceli, R 1783

Micha, JP 1657

Michael, M 462, 1554

Michel, P 1349, 1765

Michie, CO 617

Michlmayr, A 1201

Mickisch, GHJ 1309

Miki, C 787

Milde-Langosch, K 1048

Miller, CJ 929, 1136

Milne, R 597

Milne, RL 1103

Milross, CG 1510

Mimura, K 552

Minami, Y 1443

Minchinton, AI 52

Mineur, L 1349

Mirabel, X 1349

Mirasol-Lumague, MR 149

Miron, A 1103

Mishra, GD 747

Mitchell, TJ 899

Mitra, A 1229

Mitrou, PN 747

Miyagi, Y 517

Miyazaki, M 223, 469

Mizukami, Y 552

Mizumoto, Y 889

Mizuno, N 469

Mizuno, S 1644

Mlynarska, O 1269

Moccia, T 1680

Modesti, F 1835

Mogushi, K 223

Mol, L 159

Molife, LR 12, 332

Moll, I 820

Møller, H 1076

Molnár, J 178

Moore, SC 933

Moormann, AM 1736

Moranne, O 1698

Mordant, P 347

Moreau, P 1808

Moreno, V 581, 1875

Mori, M 1617

Mori, N 889

Morohoshi, T 517

Morris, DH 1760

Morrison, DS 870, 970

Morton, DG 910

Moseley, P 1794

Mottier, S 1698

Mottolese, M 1542

Moyano, S 510

Moyer, MP 975

Mudduluru, G 802

Mueller, BA 136

Mueller, BU 275

Mujcic, H 590

Mukherjee, A 1794

Mulders, P 1154

Müller, V 1048

Mulligan, AA 747

Mulligan, EA 1588

Muñoz-Martin, AJ 1524

Mupo, A 642

Murakami, S 1325

Muraro, MG 382

Murphy, G 1453, 1710

Murray, JL 1331

Murray, K 232

Nadal, A 510

Nagano, H 1617

Nagaoka, S 469

Nagase, K 1580

Nagelkerke, A 590

Nagino, M 469

Nagtegaal, ID 159

Nagy, N 930

Nakachi, K 469

Nakagawa, K 6, 36, 354

Nakahara, T 36

Nakai, Y 1644

Nakamura, M 889

Nakamura, Y 217, 517

Nakayama, Y 1325

Nambu, Y 469

Namuyenga, E 1736

Napolitano, R 1400

Narayanan, A 656

Naresh, K 265

Navarro, M 581

Négrier, S 1154

Nelson, HH 885

Nelson, M 265

Nenci, I 1835

Neri, D 827

Nesterova, A 676

Neuneier, J 1407

Newman, WG 315, 1887

Newton-Bishop, JA 1229

Ng, K-F 852

Ng, SSW 52

Nguyen, AT 291

Nicholls, AM 340

Nichols, T 209, 928

Nielsen, JE 1269

Niessen, RC 1840

Nijman, HW 685

Nikolaidis, G 1846

Nimura, Y 469

Ning, T 1085

Nishino, Y 1443

Nishiwaki, Y 6

Nitzsche, B 18

Njar, VCO 1001

Noda, K 6, 1325

Noda, S 249

Nohata, N 877

Nomura, F 223

Nomura, S 249

Norat, T 1755

Nordenstedt, H 735

Norman, AR 918

Norman, P 1448

Nortier, J 1815

Nosho, K 1025

Nsengimana, J 1229

Nuijten, M 1309

Nur, U 446

Ocker, M 18

O’Brien, LT 918

Odell, EW 1432

Odenthal, M 1407

Odero, MD 1292

Oehler, R 1201

Offit, K 1875

Ogino, S 1025

Oh, J-K 741

Ohe, M 1325

Ohkawa, S 469

Ohno, S 889

Ohtsu, T 517

Ohtsuka, M 223

Ohuchi, N 1443

Oji, Y 1255

Okada, Y 370

Okamoto, I 36, 354

Okamoto, Y 877

Okoturo, O 701

Oksuzyan, S 1128

Okugawa, Y 787

Okusaka, T 469

Olden, K 1182

Olmos, D 332

Olsen, A 730, 1755

Olsson, L 575

Olszanski, AJ 1554

O’Malley Jr, BW 1822

Omata, M 1644

Orago, AS 1736

O’Reilly, DSJ 870

Orsini, N 127

Osborn, M 1858

Oshita, F 517, 1325

Osin, P 918

Osler, M 1496

Otieno, RO 1736

Ottensmeier, C 1229

Overman, MJ 1306

Overvad, K 730, 1755

Owczarek, T 524

Owen, DA 52

Owen, LB 1671

Pabst, T 275

Paccagnella, L 332

Page, EC 918

Påhlman, L 575

Palli, D 1755

Palomo, JM 498

Pan, Y 1085

Pan, Z 1215

Pang, S-T 852

Panico, S 1755

Paoaafaite, J 1115

Papadatos-Pastos, D 607

Papadimitriou, C 1048

Papadogiannakis, N 575

Papadopoulou, MV 201

Parent, L 1562

Park, E-C 741

Park, JH 741

Park, JY 747

Park, K 845

Park, R 1076

Park, YH 845

Parker, K 656

Parlour, L 1801

Parslow, RC 1448

Pastorekova, S 1571

Patard, J-J 1698

Paul, A 101

Pauligk, C 1518

Paulin, F 232

Payne, K 315, 1887

Pchejetski, D 291

Peach, H 1229

Pecorelli, S 812

Pectasides, D 1048

Pedersen, J 411

Pedersen, LA 947

Pedretti, M 827

Pedriali, M 1835

Peet, A 1297

Peeters, M 1627

Peeters, PHM 1755

Peiffert, D 1349

Pellat-Deceunynck, C 1808

Peltola, M 708

Penault-Llorca, F 1335

Penel, N 482

Peng, G 542

Peng, Z-H 961

Penson, RT 12

Peplonska, B 1097

Perol, D 482

Perrone, M 324

Personeni, N 837

Pesatori, AC 1870

Peterlongo, P 1875

Petersen, I 505

Petit, J 1076

Petitdidier, P 1115

Pettersson, K 708

Pfeiffer, RM 1097

Pharoah, PD 668, 1137, 1875

Phillips, R 1485

Phipps, AI 1103

Piantelli, M 1835

Picelli, S 575

Pickering, LM 1149

Picton, SV 1448

Pierce, KJ 1554

Pignatelli, M 1422

Pinder, SE 94

Pirker, C 1489

Piro, G 1680

Pitiakoudis, M 1209

Pizzi, G 324

Plaz`uk, E 524

Plummer, ER 1588

Pluschnig, U 1201

Podhorska-Okołów, M 524

Pokhrel, A 1109

Polak, M 656

Pollak, MN 132, 1089

Polterauer, S 613

Polus, M 930

Ponz, M 1529

Poole, M 656

Porteous, ME 1875

Postel-Vinay, S 332

Poupon, M-F 1192

Powell, B 1229

Pozzi, F 837

Prabhakar, U 1154

Preijers, FW 1415

Preiss, JH 1173

Preissner, R 18

Pressiani, T 837

Prevost, T 701

Price, PM 201

Pries, R 1245

Proctor, MJ 870

Provenzano, E 668, 1137

Pukkala, E 1109

Pulte, D 149

Punt, CJA 159, 1415

Purdie, CA 475

Puumala, SE 136, 1724

Pwu, R-F 1773

Qian, Z 567

Qin, X 1154

Qiu, G-Q 961

Quaresma, M 446

Querzoli, P 1835

Quideau, S 61

Quinlan, P 475

Quinn, JA 401

Quintero, G 1536

Rachedi, F 1115

Rachet, B 446

Rahhal, J 613

Rahmathulla, G 498

Raimondi, E 1835

Rajaraman, P 727

Rajeshkumar, NV 649

Raji, OY 423

Rajpert-De Meyts, E 1269

Rakha, E 1794

Ranchère-Vince, D 165

Randers-Pehrson, G 1263

Raoul, JL 1349

Raphael, S 1432

Rashid, A 115

Rass, K 820

Rassl, DM 1710

Rautalahti, M 1109

Ray-Coquard, I 165, 482

Raynaud, F 1313

Razavi, D 171

Rebolj, M 310

Reboredo, M 1536

Rech-Weichselbraun, I 1201

Redaniel, MT 149

Reddy, N 1822

Reid, AHM 332

Reinacher-Schick, A 1407

Reinsberg, SA 52

Reinthaller, A 613

Remenar, E 1173

Ren, MQ 1066

Renshaw, C 1076

Resnick, MB 885

Rettenmaier, CR 1657

Rettenmaier, NB 1657

Revaud, D 591, 1475

Reynaert, C 171

Reyners, AKL 757

Reynolds, CP 1369

Reynolds, P 136

Rezai, K 560

Riboli, E 1755

Richards, CH 1356

Richards, M 1448

Richter, CE 812

Rickenbach, M 416

Riesco-Martinez, M 1524

Rijkaart, DC 939

Rimassa, L 837

Rinaldi, R 1835

Rinaldi, S 1755

Rintoul, RC 1710

Rio, E 1349

Rioux-Leclercq, N 1698

Risk, JM 1846

Rivans, I 617

Rives, M 1349

Rizzello, F 975

Robé, A 715

Roberts, JD 1182

Robinson, CG 498

Robson, W 701

Rochaix, P 61

Rochford, R 1736

Rodriguez, J 1529

Rodríguez, L 1755

Rodwell, SA 747, 1755

Rody, A 1048

Roesler, MA 1724

Rogers, KD 1034

Rogers, M 101

Romaguera, D 1755

Romero, C 1536

Roobol, MJ 708

Roos, G 1255

Rose, BR 1510

Rose, S 597

Rosenbaum, PF 1736

Rosenthal, MA 462

Ross, JA 1724

Rossi, J-F 1154

Rossoni, G 837, 837

Rotstein, S 1706

Rotunno, M 1870

Roux, M 715

Rozanska, AL 1588

Rozendaal, L 939

Rubagotti, M 1870

Rubbi, CP 186

Rubino, M 1182

Rubio-Viqueira, B 649

Rudek, MA 649

Ruiz-Garcia, A 993

Rusciani, A 1562

Russell, PJ 1008

Russo, A 1305

Russo, G-L 642

Russo, M 642

Rutegård, M 735

Rutgers, EJT 1788

Rutherford, TJ 812

Ryan, MC 676

Sabnis, G 1001

Sabourin, JC 1765

Sage, EK 1692

Saigusa, S 787

Saijo, N 6

Saini, S 256

Saintigny, P 560

Saito, H 1325

Sakala, M 617

Sakurai, D 877

Sala, C 827

Salek, RM 1297

Salgado, M 1536

Salmon, I 930

Sánchez, M-J 1755

Sandberg, P-O 1706

Sandin, R 1307

Sansbury, LB 1453

Santella, RM 1263

Santin, AD 812

Santini, D 1305

Santoro, A 837

Sargent, A 209, 928

Sarker, D 607

Sartori, G 1019

Sasahira, N 1644

Sasaki, T 1644

Sasamata, M 36

Sato, E 552

Sauer, C 196

Saunders, NA 73

Savage, CJ 708

Scandolera, A 1562

Scardino, PT 708

Scarlett, CJ 391

Scarpa, A 391

Schache, AG 1846

Schadendorf, D 820

Scharenborg, NM 1415

Schässburger, K-U 1706

Schatzkin, A 933, 1453

Schayowitz, A 1001

Scheffler, M 693

Scheithauer, W 1318

Schelz, Z 178

Schmid, K 622

Schmid, S 275

Schmidt, M 1048

Schmiedel, S 931

Schmiegel, W 1407

Schmitt, CA 505

Schmitt, M 663

Schnell, R 1407

Schoemaker, MJ 1760

Scholz, M 1518

Schrader, M 18

Schröder, FH 708

Schultz, MK 796

Schulze, URJ 685

Schüz, J 1128

Schwartz, PE 812

Sciuto, R 324

Scoazec, J-Y 165

Scudamore, CH 52

Sebbag, J 1115

Seebacher, V 613

Sehlen, S 1489

Seki, N 877

Sekine, S 1057

Selby, PJ 101, 1649

Selle, F 560

Senellart, H 1349

Seow, A 1093

Seppä, K 1109

Servent, V 772

Sesboüé, R 1765

Severi, G 411, 668, 1137

Seynaeve, C 765

Shabaruddin, FH 315, 1887

Shah, A 446

Shahryari, V 256

Shan, L 1115

Shankar, A 1093

Shannon, BA 411

Sharma, S 1066

Sharman, JA 1422

Shaw, HM 332

Shaw, RJ 1846

Shebl, FM 115

Sheen, I-S 954

Shehata, M 1794

Shen, M 727

Shen, M-C 115

Shenton, G 1448

Shepherd, N 1034

Sherman, ME 1097

Shibata, T 6

Shida, T 223

Shiels, PG 899

Shim, Y-M 845

Shima, K 1025

Shimada, K 1057

Shimizu, C 297

Shimizu, H 223

Shin, A 741

Shinohara, T 517

Shipley, MJ 747

Sibson, DR 186

Sieghart, W 622

Sieuwerts, AM 1284

Sigal-Zafrani, B 1192

Silasi, D-A 812

Silverman, DT 727

Sim, S 1649

Simpson, JM 1502

Sims, AH 401

Sinclair, R 411

Singh, AP 1671

Singh, S 1671

Sinha, R 12

Sirab, N 591, 1475

Sitaram, RT 1255

Sivridis, E 1209

Skakkebæk, NE 1269

Slachmuylder, J-L 171

Slotman, BJ 29

Smallwood, J 1229

Smedh, K 575

Smigova, A 201

Sminia, P 29

Smith, ES 401

Smith, G 597

Smith, LM 676

Smith, MR 1783

Smith, P 265

Smith, PC 1040

Smith, RC 391

Snijders, PJ 939

Snyder, K 132, 1089

Sogawa, K 223

Solari, V 593

Soldan, K 209, 928

Sommerville, S 73

Sondak, VK 1548

Søndergaard, F 947

Sørensen, HT 947

Sorensen, M 12

Soria, J-C 347

Soria, JC 993

Southey, MC 411, 1103

Spagnoli, G 382

Spagnuolo, C 642

Span, PN 590

Spears, M 663

Spector, LG 136

Spencer, EA 747

Sperduti, I 324, 1542

Spicer, J 332

Spieth, K 820

Spinetti, G 1422

Spisni, E 975

Spitz, MR 423

Spitzer, D 196

Sposto, R 1369

Spoto, C 1305

Srivastava, SK 1671

Staib, L 1163

Stalpers, LJA 29

Standfuß, C 505

Stanifer, ML 885

Stanimirovic, D 1606

Stanley, P 1229

Stark, D 1448, 1485

Stasinopoulou, G 1755

Stearns, V 291

Stebbing, J 265, 291

Steenbergen, RDM 29

Steger, G 1201

Stein, J 1597

Stephen, AM 747

Stering, K 663

Stewart, JS 1173

Stoeber, K 701

Stoeckigt, D 1548

Stolzenberg-Solomon, RZ 1089

Stone, N 1034

Stopatschinskaja, S 1518

Stoppa-Lyonnet, D 1192

Storniolo, AM 291

Strachan, MWJ 617

Stram, DO 120

Stratford, IJ 201

Strauss, L 196

Stresemann, C 820

Strillacci, A 975

Ströbel, P 196

Su, W-P 1343

Su, Y 1571

Sucker, A 820

Sugar, R 1313

Sugiyama, H 1255

Suh, SO 256

Sumba, PO 1736

Sun, H-C 961

Sun, M 1085

Sung, J 741

Sutherland, MK 676

Suttie, S 232

Suzuki, H 877

Swanson, J 1122, 1128

Swanton, C 155, 607, 1139

Swerdlow, AJ 1729, 1760

Syed, N 265

Szatmári, I 178

Tada, M 1644

Tagliabue, G 1755

Takakura, M 889

Takano, S 223

Takashima, T 249

Takeda, K 6

Takeda, Y 1617

Takezawa, K 36, 354

Takhar, KS 52

Talwar, D 870

Tamura, K 297

Tamura, T 6

Tan, AC 649

Tan, BA 1831

Tan, E 430

Tanaka, H 223

Tanaka, K 787

Tanaka, Y 256

Tanemura, M 1617

Tang, C-H 1773

Tang, H-M 961

Tanioka, M 297

Tarpin, M 1562

Tateishi, K 1644

Taylor, AP 82

Taylor, J 265

Taylor, KJ 663

Taylor, MA 776

Tedesco, I 642

Teerenstra, S 159

Telfer, BA 201

Tempfer, C 613

ten Hoor, KA 685

Tenesa, A 575, 1875

Teng, Y 1066

Terracciano, L 382

Teuri, J 1115

Theodoratou, E 1875

Theriault, RL 1331

Thevenard, J 1562

Thiel, A 1245

Thomas, E 715

Thomas, HD 1588

Thomas, K 1149

Thompson, A 232

Thompson, AM 475

Thompson, D 101, 1649

Thomson, JA 954

Tie, J 1885

Tillier, C 993

Timmer-Bonte, JNH 1362, 1637

Tisch, U 542

Tittarelli, A 1128

Tixier, H 1335

Tjønneland, A 730, 1755

Tjornelund, J 12

To, SST 362

Tobar, N 1040

Tobinai, K 1580

Todeschini, P 812

Togawa, O 1644

Toiyama, Y 787

Tomasi, V 975

Tomer, KB 1182

Tomimaru, Y 1617

Tomlinson, IP 1875

Tomokuni, A 1617

Tonini, G 1305

Tornillo, L 382

Törnqvist, A 575

Torsello, A 1542

Tóth, D 178

Toy, J 1076

Trenkle, T 1245

Triantafyllou, A 1846

Trichopoulou, A 1755

Tsai, H-J 1343

Tsilidis, KK 1755

Tsuchiya, E 217

Tsuda, H 297, 1057

Tsuji, I 1443

Tsuji, S 517

Tsujino, T 1644

Tsukioka, S 354

Tsuura, Y 517

Tubiana-Hulin, M 482

Tumino, R 1755

Tumolo, S 1542

Turashvili, G 668, 1137

Turnbull, JE 593

Tuthill, M 265

Tweddle, DA 1588

Uchida, J 354

Ueno, NT 927, 1331

Ugolini, D 885

Ugorski, M 524

Umeshita, K 1617

Unoki, M 217

Urbas, P 1548

Uson, M 649

Valera, A 510

Valerii, MC 975

Valero, V 1331

Valery, PC 1663

Valladares, M 581

Valle, JW 315, 1887

van Agthoven, T 1284

van Beijsterveldt, LEC 987

van Breda, SG 1749

van Dam, P 532

van De Rakt, M 1415

van den Berg, J 29

van den Weyngaert, D 1173

Van der Auwera, I 532

van der Gaast, A 987

van der Graaf, WTA 1637

van der Meide, WF 29

van der Veldt, AA 757

van der Zee, AGJ 685

van Duijnhoven, FJB 1755

van Erp, NP 757

van Fessem, MA 765

van Gelder, T 765

van Gils, CH 1755

van Guelpen, B 1755

van Herpen, CML 1173, 1362, 1637

van Kemenade, FJ 939

van Krieken, JHJM 159

van Laar, M 1448

Van Laar, RK 1852

Van Laere, SJ 532

Van Laethem, J-L 930

van Lenthe, FJ 1742

Van Marck, E 1627

van Nifterik, KA 29

van Nimwegen, M 629

van Schaik, RHN 765

van Spronsen, D-J 1415

Van, JT 772

van’ t Veer, LJ 1788

Vasey, PA 597

Vassiliou, V 925

Vasudev, NS 101, 1649

Vázquez, I 1292

Veltman, JD 629

Venkatasubramanian, R 486

Vennarecci, G 1542

Vercillo, MS 1221

Veres, T 1606

Verheijen, RH 939

Verhoeven, RHA 1462

Vermeulen, PB 532

Vermorken, JB 1173, 1627

Verpelli, C 827

Verweij, J 987, 1362

Verweij, W 939

Vicente, C 1292

Vickers, AJ 708

Vidal, L 12, 332

Vigneau, C 1698

Villaroel, MC 649

Vincent, TJ 1122

Vincent-Salomon, A 1192

Vincenzi, B 1305

Vinceti, M 1128

Vineis, P 1755

Virtamo, J 132, 1089

Vlahovic, G 1554

Vlatković, N 186

Vogelbaum, MA 498

Volpe, S 642

Von Behren, J 136

von Holst, S 575

von Schweinitz, D 43

Vonderhaar, BK 1097

Vormoor, J 439

Vose, JM 1783

Vowler, SL 1710

Vulto, AG 765

Wada, H 1617

Wadström C, 1706,

Wagner, A 1840

Wakasa, K 249

Walker, C 1229

Walters, S 446

Walzer, S 1309

Wang, B-S 115

Wang, E 1870

Wang, F 567

Wang, H 1306, 1822

Wang, J-H 954

Wang, Q 961

Wang, T-E 954

Wang, X 1085

Wang, X-L 961

Wang, Z 1085

Wang-Peng, J 954

Ward, L 1663

Warnakulasuriya, S 303

Warren, N 430

Warren, WH 1221

Wasylyk, B 715

Wasylyk, C 715

Watanabe, K 6

Watanabe, M 552

Watanabe, T 1580

Waxman, J 291

Webb, E 1875

Weber, GF 861

Weber, J 1548

Wedekind, LE 29

Wei, J 567

Wei, W 910

Weihrauch, MR 1407

Wen, Y-G 961

Weng, W-H 852

Werzowa, J 622

Wesseling, J 1788

Wessels, JAM 757

West, CM 929, 1136

West, DW 1103

Weyler, J 1627

White, DE 1380

White, IR 747

Whittemore, AS 1103

Wickens, M 1710

Wiegel, T 1163

Wiksell, H 1706

Wildiers, H 1815

Wilkens, L 275

Wilkinson, RA 918

Wilks, A 597

Williams, GH 701

Williams, KJ 201

Wilson, G 209, 928

Wilson, M 1297

Wilson, SR 391

Win, AK 1875

Winkler, H 987

Wirtz, RM 1048

Wischhusen, J 693

Witney, TH 1400

Witzel, I 1048

Wojciechowski, T 1657

Wolff, A 196

Wolff, RA 1306

Wolk, A 127

Wollenberg, B 1245

Wollenschlaeger, A 701

Wong, K-Y 1093

Wong, MQ 52

Wood, SL 101

Woods, L 446

Woolgar, JA 1846

Wouters, B 590

Wu, C-F 852

Wu, J 567

Wu, L 1263

Wu, M 1215

Wunsch Filho, V 1128

Wuyts, W 1627

Wyld, DK 597

Wynendaele, W 1815

Xia, J 961

Xiao, W 1008

Xu, A 1263

Xu, Z 567

Xue, A 391

Xue, X 567

Yagioka, H 1644

Yamada, K 517, 1057, 1325

Yamada, Y 36

Yamamoto, K 1644

Yamanaka, K 36

Yamashita, K 1325

Yamashita, T 1822

Yan, D-W 961

Yang, P-C 1144

Yang, T 1215

Yang, T-S 954

Yang, XR 1097

Yang, Y 1215

Yang, Y-X 961

Yaniv, I 1597

Yao, J 567

Yap, TA 332

Yapp, DTT 52

Yashima, Y 1644

Yashiro, M 249, 1182

Yasuda, H 787

Yataghène, Y 1349

Yates, EA 593

Yeadon, T 597

Yen, C-J 1343

Yin, D 332

Yip, SP 362

Yokoe, T 787

Yokosuka, O 223

Yokoyama, A 6

Yonemori, K 297

Yoshida, M 1057

Yoshidome, H 223

Yoshimura, K 297

Yoshitomi, H 223

You, J-F 1840

You, S-L 1773

Youlden, DR 1663

Younes, A 1783

Yu, C 676

Yu, K 115

Yu, MC 1093

Yu, S-L 1144

Yu, Z 1263

Yuan, Z 987

Yun, EH 741

Yung, A 52

Yung, YMB 362

Zakrzewicz, A 18

Zaman, MS 256

Zarate, R 1529

Zavada, J 1057

Zelenetz, AD 1783

Zeuli, M 1542

Zhang, B-H 115

Zhang, J 1215

Zhang, S 1085

Zhao, M 649

Zhao, Y 1085, 1870

Zhou, C-Z 961

Zhou, H 1263

Zhou, W 1215

Zhou, X-L 1875

Ziegler, K 693

Zimmermann, C 1477

Zino, S 899

Zipp, K 693

Ziprin, P 1858

Zironi, S 1019

Zlobec, I 382

Zoccoli, A 1305

